# Analysis of plant pararetrovirus promoter sequence(s) for developing a useful synthetic promoter with enhanced activity in rice, pearl millet, and tobacco plants

**DOI:** 10.3389/fpls.2024.1426479

**Published:** 2024-08-06

**Authors:** Khushbu Kumari, Tsheten Sherpa, Nrisingha Dey

**Affiliations:** ^1^ Division of Plant Biotechnology, Institute of Life Sciences, Bhubaneswar, Odisha, India; ^2^ Regional Centre for Biotechnology, National Capital Region Biotech Science Cluster, Faridabad, Haryana, India

**Keywords:** synthetic promoter, monocot, dicot, GUS, plant pararetrovirus

## Abstract

Promoters are one of the most important components for many gene-based research as they can fine-tune precise gene expression. Many unique plant promoters have been characterized, but strong promoters with dual expression in both monocot and dicot systems are still lacking. In this study, we attempted to make such a promoter by combining specific domains from monocot-infecting pararetroviral-based promoters sugarcane bacilliform virus (SCBV) and banana streak virus (BSV) to a strong dicot-infecting pararetroviral-based promoter mirabilis mosaic virus (MMV). The generated chimeric promoters, MS, SM, MB, and BM, were tested in monocot and dicot systems and further validated in transgenic tobacco plants. We found that the developed chimeric promoters were species-specific (monocot or dicot), which depended on their respective core promoter (CP) region. Furthermore, with this knowledge, deletion-hybrid promoters were developed and evaluated, which led to the development of a unique dual-expressing promoter, MSD3, with high gene expression efficiency (GUS and GFP reporter genes) in rice, pearl millet, and tobacco plants. We conclude that the MSD3 promoter can be an important genetic tool and will be valuable in plant biology research and application.

## Introduction

Bioengineering of plants using synthetic biology to combat diverse problems such as food security, climate change, and overpopulation is essential for a sustainable future (Shih, 2018). Plant synthetic biology has opened many paths to utilize plants in different ways, such as bio-farming for producing pharmaceuticals or fuels, multi-stress resistant plants, bio-fortified crops, biosensors, etc (Liu and Stewart, 2015; Yang et al., 2022a; Wang and Demirer, 2023; Joshi and Hanson, 2024). One key component of synthetic biology is the synthetic promoter(s) responsible for fine-tuning the gene(s) that are being expressed. During the last three decades, several native and synthetic promoters have been characterized to express foreign genes in plants efficiently. Plant pararetroviruses, which are plant-infecting viruses, have been a rich source of strong, constitutive plant promoters, especially in dicotyledonous plants where several efficient promoters from the cauliflower mosaic virus (CaMV), figwort mosaic virus (FMV), mirabilis mosaic virus (MMV), horseradish latent virus (HRLV), dahlia mosaic virus (DaMV), and strawberry vein banding virus (SVBV) have been characterized (Odell et al., 1985; Maiti et al., 1997; Dey and Maiti, 1999; Pattanaik et al., 2004; Sahoo et al., 2015; Khan et al., 2018; Sherpa et al., 2023). Most promoters reported during this period are mainly dicot-specific, while effective monocot-specific promoters are still scarce (Zhang et al., 2016; Cai et al., 2020). Mostly, endogenous ubiquitin and actin-based promoters are used for monocot transformation. Such native promoters are usually larger and prone to the host’s innate signaling processes (Dey et al., 2015; Liu and Stewart, 2016; Yasmeen et al., 2023). There are only a few reports that describe the characterization of efficient pararetroviral-based monocot promoters such as the rice tungro bacilliform virus (RTBV) (He et al., 2002; Gupta et al., 2021), commelina yellow mottle virus (CoYMV) (Medberry and Olszewski, 1993), sugarcane bacilliform virus (SCBV) (Davies et al., 2014), and banana streak virus (BSV) (Remans et al., 2005). Studies have shown that the dicot-infecting pararetroviral promoters from CaMV, FMV, MMV, and HRLV are highly active in dicots, and the monocot-infecting pararetrovirus promoters from SCBV and BSV are highly active in the monocots (Bhattacharyya et al., 2002; Remans et al., 2005; Gao et al., 2017; Cai et al., 2020; Sherpa et al., 2023).

It has been observed that most of the strong dicot promoters are not efficiently expressed in monocots, and conversely, strong monocot promoters are not efficiently expressed in dicots (Christensen et al., 1992; Gupta et al., 2001; Park et al., 2010). This variation might result from species-specific differences in genomic GC content and transcription factors (Jores et al., 2021). Studies in the core promoter (CP) region of dicot and monocot promoters found that low GC-containing CPs were fourfold stronger than GC-rich promoters in dicot plants. In contrast, in the case of the monocot CPs, the GC content did not significantly impact promoter activity (Jores et al., 2021). Furthermore, different transcription factor binding sites in monocot and dicot behaved differently between them. For example, promoters containing motifs for HSFs (heat shock factors) were more active in monocots than dicots, and promoters containing TCP TF motifs were more active in dicots than monocots (Jores et al., 2021). Therefore, separate promoters are being used to transform monocot and dicot plants. In dicot plants, the most extensively used promoter is a viral-based promoter called CaMV35S from the CaMV, and for monocots, the most extensively used promoter is the endogenous ubiquitin promoter of maize called ZmUbi1. Although it has been found that the CaMV35S promoter is active in monocot plants, its overall strength is four- to fivefold lower than its activity in dicots and is also comparatively lower than the ZmUbi1 promoter (Christensen et al., 1992; Cai et al., 2020; Jores et al., 2021). There is still no individual promoter with dual usage in both monocot and dicot plants for high-level gene expression.

In context to the above, we assume that a specific domain of the dicot plant-expressing promoters from pararetroviruses in combination with a specific region of monocot-expressing promoters could be an important genetic resource for developing effective synthetic monocot–dicot-expressing chimeric promoters. Therefore, we attempted to develop a common promoter for monocot and dicot plants in the present study. Accordingly, we combined strong promoter fragments from monocot-infecting pararetroviruses, SCBV (coordinate: −770 to +69) and BSV (coordinate: −1,150 to +154), to a strong promoter fragment from a dicot-infecting pararetrovirus, MMV (coordinate: −297 to +63), individually, for developing chimeric promoters, namely, BM, MB, SM, and MS, and their activities in both dicot and monocot systems have been evaluated. Furthermore, a strong promoter MSD3 was developed by the conjugation of MMV, −297 to −38, to SCBV, −340 to +69, which showed high gene expression capability in rice, pearl millet, and tobacco plants, and it could be a useful addition in gene-based plant biotechnology research and application.

## Materials and methods

### Materials

The SCBV-Ireng Maleng isolate (851 bp; accession number AJ277091) and BSV-Cavendish isolate (1,304 bp; accession number AF215815) sequences were synthesized from GenScript Biotech, New Jersey, USA [Order ID: XI-14-SM-/2018-19 (11)/1565/ILS]. Dr. I.B. Maiti, University of Kentucky, USA, kindly provided the genetic material for MMV, the CaMV-full length transcript promoter (CaMV35S), the maize ubiquitin-1 promoter (ZmUbi1), and seeds for *Nicotiana tabacum* and *Nicotiana benthamiana* plants. The seeds for *Oryza sativa* cv. IR64 were obtained from ICAR-National Rice Research Institute (NRRI), Cuttack, and pearl millet seeds from the International Crops Research Institute for the Semi-Arid Tropics (ICRISAT), Hyderabad. All restriction enzymes, T4 DNA ligase, Klenow Fragment, and *Taq* DNA Polymerase were purchased from Thermo Fisher Scientific (USA), and chemicals such as MS salts, MUG, β-mercaptoethanol, DNA ladders, and X-Gluc were purchased from Sigma-Aldrich (USA). General chemicals such as CaCl_2_, sucrose, glucose, KCl, MES, and EDTA were purchased from HiMedia Laboratories Pvt Ltd (India).

### Construction of plant expression vectors

#### Developing mother promoter constructs

The SCBV (−770 to +69; 839 bp), BSV (−1,150 to +154; 1,304 bp), MMV (−297 to +63; 360 bp), and CaMV35S (−940 to +27; 967 bp) were cloned into *EcoRI* and *HindIII* sites in the pUC119 vector to develop pUSCBV, pUBSV, pUMMV, and pUCaMV35S vectors. In the case of the ZmUbi1 promoter (−899 to +1,093; 1,992 bp), the internal *EcoRI* site at coordinate +492 to +498 was modified by digestion with *EcoRI*, followed by end-filling using *Klenow* fragment, and self-ligation with T4 DNA ligase. The resultant ZmUbi1 promoter was cloned into the pUC119 vector, leading to the development of the pUZmUbi1 vector. All these above pUC119 clones were sub-cloned into a plant expression vector pKYLX71GUS (Schardl et al., 1987), which contained the *GUS* reporter gene, leading to the development of pKSCBVGUS, pKBSVGUS, pKMMVGUS, pKCaMV35SGUS, and pKZmUbi1GUS.

#### Developing hybrid promoter constructs

The upstream activation sequence (UAS) fragments of SCBV (SUAS; −434 bp to −153 bp; 282 bp), BSV (BUAS; −1,150 to −33; 1,117 bp), and MMV (MUAS; −297 to −38; 259 bp) were PCR amplified using respective primers (**Supplementary Table S1**) containing the *EcoRI*/*HincII* site in the 5′ region and the *SmaI*/*HindIII* site in the 3′ region and subsequently cloned into the pUC119 vector to generate pUSUAS, pUBUAS, and pUMUAS respectively. Likewise, the CP domain of SCBV (−770 to +69), BSV (−1,150 to +154), and MMV (−297 to +63) were PCR amplified with respective primer pairs (**Supplementary Table S1**) and cloned into pUC119 to generate pUSCP, pUBCP, and pUMCP vectors, respectively. Finally, the plasmid pUSCP, pUBCP, and pUMCP were double digested with *HincII* and *HindIII* enzymes, and the insert *HincII*-SCP-*HindIII* was cloned into the *SmaI* and *HindIII* sites of the pUMUAS vector leading to the development of the pUMUASSCP construct. Similarly, the insert *HincII*-MCP-*HindIII* was cloned into the *SmaI* and *HindIII* sites of the pUSUAS hybrid clone, leading to the development of the pUSUASMCP hybrid clone. A similar strategy was followed for developing pUBUASMCP and pUMUASBCP hybrid vectors. Finally, the chimeric promoters MUASSCP (MS), SUASMCP (SM), BUASMCP (BM), and MUASBCP (MB) were sub-cloned into the pKYLX71GUS expression vector in *EcoRI* and *HindIII* sites, leading to the development of pKMSGUS, pKSMGUS, pKBMGUS, and pKMBGUS.

#### Developing deletion-hybrid promoter constructs

Furthermore, we made four deletion constructs from SCBV, namely, SD1 (−640 to +69), SD2 (−430 to +69), SD3 (−340 to +69), and SD4 (−140 to +69), and five deletion constructs from BSV promoters, namely, BD1 (−1,013 to +154), BD2 (−867 to +154), BD3 (−722 to +154), BD4 (−583 to +154), and BD5 (−433 to +154), using respective primers (**Supplementary Table S2**), and the generated fragments were cloned into pUC119. The resultant clones (a total of nine) were designated as pUSD1, pUSD2, pUSD3, pUSD4, pUBD1, pUBD2, pUBD3, pUBD4, and pUBD5, and sequences were confirmed. Subsequently, the promoters were cloned individually to the pUMUAS vector by following a similar strategy as above, and the generated clones were named pUMUASSD1, pUMUASSD2, pUMUASSD3, pUMUASSD4, pUMUASBD1, pUMUASBD2, pUMUASBD3, pUMUASBD4, and pUMUASBD5. Finally, the deletion-hybrid promoters MUASSD1 (MSD1), MUASSD2 (MSD2), MUASSD3 (MSD3), MUASSD4 (MSD4), MUASBD1 (MBD1), MUASBD2 (MBD2), MUASBD3 (MBD3), MUASBD4 (MBD4), and MUASBD5 (MBD5) were sub-cloned into plant expression vector pKYLX71GUS, leading to the development of pKMSD1GUS, pKMSD2GUS, pKMSD3GUS, pKMSD4GUS, pKMBD1GUS, pKMBD2GUS, pKMBD3GUS, pKMBD4GUS, and pKMBD5GUS.

Additionally, *GFP* reporter gene was cloned into vectors pKCaMV35SGUS, pKZmUbi1GUS, pKMSD3GUS, and pKMBD4GUS by replacing *GUS* gene, leading to the development of pKCaMV35SGFP, pKZmUbi1GFP, pKMSD3GFP, and pKMBD4GFP vectors.

### Fluorometric GUS assay

The biochemical-based fluorometric GUS assay was performed by following the method described by Jefferson et al. (1987). Briefly, the samples were crushed and resuspended into GUS extraction buffer containing 50 mM NaPO_4_, 1 mM EDTA, 10 mM DTT, 0.1% Triton-X, and 0.1% SDS. The mixture was centrifuged, and the supernatant was collected and transferred to 1 mM 4-methylumbelliferyl-β-D-glucuronide hydrate (MUG) solution. At every time interval of 0, 10, and 20 min, the solution was transferred to 0.2 M Na_2_CO_3_ solution (stop solution), and fluorescence was measured. Finally, the total protein content was measured using the Bradford reagent and used to normalize the total GUS activity for each sample.

### Histochemical staining

For detection of GUS expression in tissue samples, an X-Gluc solution containing 0.3% X-Gluc (5-bromo-4-chloro-3-indolyl-β-d-glucuronic acid), 50 mM NaPO_4_, 10 mM EDTA, and 0.01% Tween-20 was prepared. The tissue samples were dipped into this X-Gluc solution and kept at 37°C for 12 h. Next, the samples were kept in 70% ethanol until the chlorophyll was removed and photographed.

### Transient analysis of promoters

All promoter constructs cloned into the pKYLX71 vector were transformed into *Agrobacterium tumefaciens* GV3101 using the freeze–thaw technique (Chen et al., 1994), and the positive agrobacterium colony was then infiltrated into tobacco leaves and rice and pearl millet seedlings following an earlier protocol (Sethi et al., 2022; Sherpa et al., 2023). Briefly, *N. tabacum* and *N. benthamiana* were germinated into the soil and grown for 4–6 weeks in greenhouse conditions (16/8 h light/dark cycle, temperature: 22–28°C, humidity: 70%–75%). The positive agrobacterium colonies were grown overnight and resuspended in an agro-infiltration buffer containing 20 mM Na_3_PO_4_, 50 mM MES, 0.1 mM acetosyringone, and 27 mM D-glucose. Finally, the agrobacterium suspension was infiltrated using a needleless syringe in healthy tobacco leaves’ abaxial side.

In the case of rice and pearl millet infiltration, the seeds were germinated in Petri plates containing wet-blotting paper for 2–4 days in the dark. After germination, the plates with seedlings were kept in greenhouse conditions for 7–14 days. The positive agrobacterium colony carrying the promoter clones were resuspended individually into liquid infiltration buffer containing 4 g/L MS salt, 200 mM glucose, 200 mM sucrose, 40 mM KCl, 42 mM MgCl_2_, 150 µM acetosyringone, and 0.01% Silwet. The seedlings were dipped into the agro suspension and infiltrated using a vacuum at 0.933 bar pressure for 10 min.

The GUS activity was measured from agro-infiltrated tobacco leaves, rice seedlings, and pearl millet seedlings after 48 h of post-infection using a fluorometric GUS assay described above.

For GFP activity analysis, we followed earlier established protocols (Sethi et al., 2022; Sherpa et al., 2023). Briefly, tobacco leaves and rice seedlings were agro-infiltrated using pKCaMV35SGFP, pKZmUbi1GFP, pKMSD3GFP, and pKMBD4GFP containing agrobacterium clones as described above and were kept for 5 days in greenhouse conditions post-infection and imaged under UV light using the Gel Doc XR + System (Bio-Rad). The GFP intensity of the images was measured using ImageJ software (Schneider et al., 2012).

### Promoter activity assay in tobacco, rice, and pearl millet callus

The callus for tobacco, rice, and pearl millet was generated in tissue culture following standard protocols. For tobacco callus generation, a standard protocol for the leaf disc transformation method was followed using wild-type leaves of *N. tabacum* (Horsch et al., 1989). For rice callus generation, *O. sativa* L. cv. IR 64 seeds were used to generate embryogenic callus following a standard protocol (Majumder et al., 2021). Briefly, the seeds were sterilized, and the embryo was dissected and placed on callus induction media (CIM; MS media, 300 mg/L casein hydrolysate, 2.8 g/L proline, 2 mg/L 2,4-dichlorophenoxyacetic acid, 30 g/L sucrose, and 1% agar, pH 5.8) for 15 days at 25°C in completely dark conditions. After 15 days, the calli were subcultured on fresh CIM media for 10 days, and the generated calli were used for further assay. In the case of pearl millet callus, a previously established protocol was followed where the seeds post-sterilization were plated on CIM (MS media, 440 mg/L CaCl_2_, 30g/L sucrose, 3.0 mg/L 2,4-dichlorophenoxyacetic acid, 0.5 mg/L kinetin, and 0.8% agar, pH 5.8) for 15 days at 25°C in complete darkness and sub-cultured in fresh media for 10 days (Chanwala et al., 2024; Jha et al., 2024). The generated callus was then used for further analysis.

All the promoters were transformed into the generated callus from tobacco, rice, and pearl millet by immersing them in agrobacterium culture for 20 min with constant shaking at 100 rpm. The calli were then dried on blotting paper, transferred to their respective CIM, and kept for 2 days in the dark. After 2 days, the calli were subcultured on fresh media supplemented with 300 mg/L kanamycin and 1 g/L cefotaxime and kept for 15–20 days in a light/dark cycle at 25°C. Finally, the calli were crushed using liquid nitrogen, and the fluorometric GUS assay was performed.

### Development of transgenic tobacco plants

The hybrid promoter containing vectors, pKBMGUS, pKMBGUS, pKSMGUS, and pKMSGUS, along with pKCaMV35SGUS were transformed into *Agrobacterium tumefaciens* LBA4404, and the positive agro colony was selected for infecting the tobacco leaf following the standard protocol (Horsch et al., 1989). A total of 10 transgenic lines were selected based on gene integration assay, where genomic DNA from plants was isolated, and PCR amplified using *nptII*, *rbcSE9*, and *GUS* gene-specific primers (**Supplementary Table S3**). Next, the seeds from the T_0_ generation were harvested, followed by segregation analysis. Briefly, the seeds were germinated in 0.5× MS medium plates containing 300 mg/L kanamycin and were kept at 4°C overnight and transferred to the tissue culture room under a light–dark cycle (16 h light/8 h dark). The plates were kept for 21 days until the seeds were in two leaf stages. The Kan^R^ (resistant) and Kan^S^ (sensitive) seedlings were counted, and a chi-square analysis was carried out. The lines that followed the best 3:1 segregation ratio with a chi-square value of <1.5 (*p* ≤ 0.05) were selected for further analysis and grown till T_2_ generation.

### Statistical analysis

All the procedures were performed three times, and the mean of the independent experiments was measured and presented with their respective standard deviation. Statistical significance was calculated using Student’s *t*-test, and the level of significance was represented as asterisks where *p ≤ 0.05; **p ≤ 0.01; ***p ≤ 0.001.

## Results

### Comparative transient analysis of developed promoters

**Figure 1:**
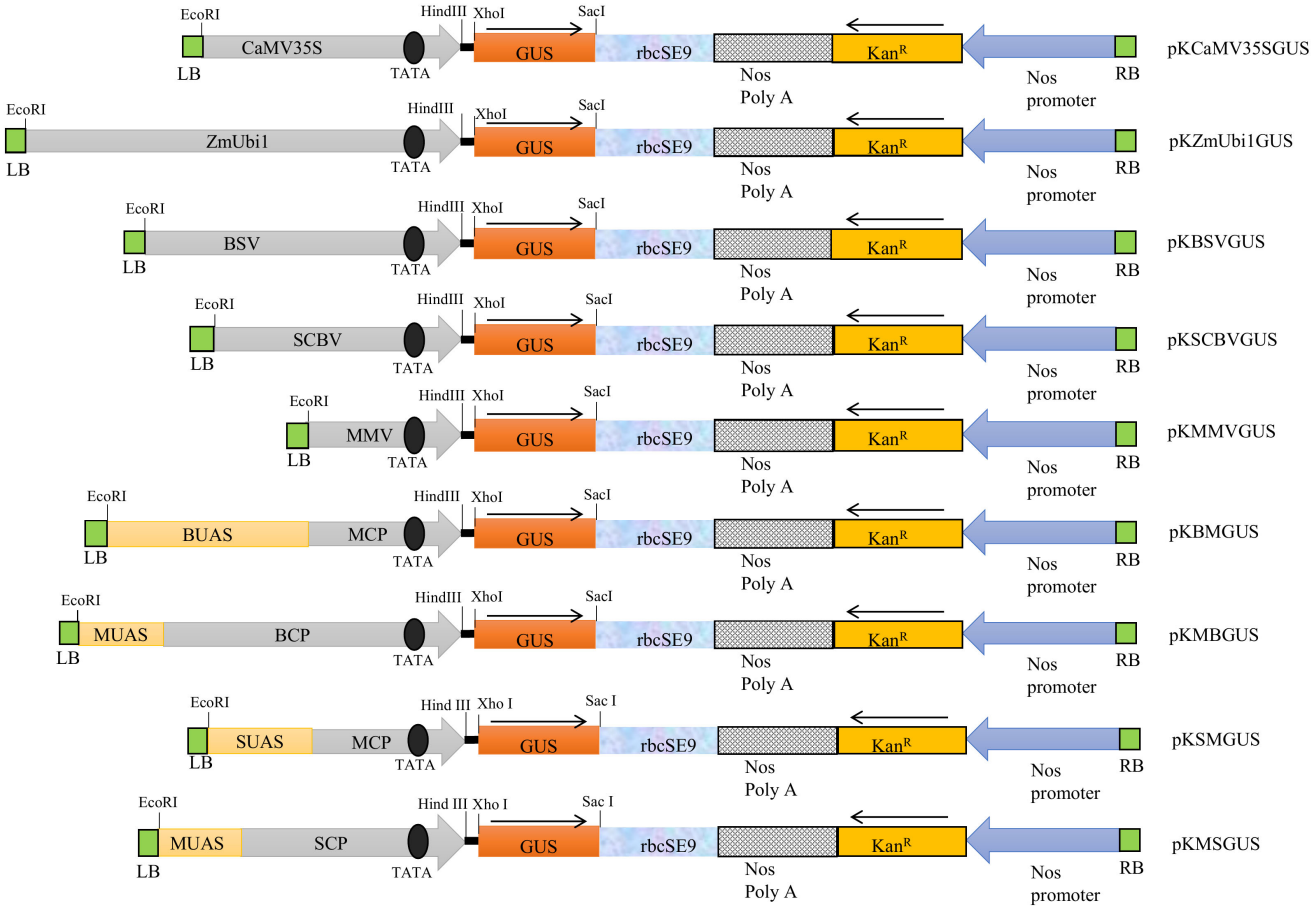
The schematic representation of the T-DNA region of binary vector pKYLX71 harboring promoters, namely, CaMV35S, ZmUbi1, BSV, SCBV, MMV, BM, MB, SM, and MS, coupled to GUS reporter gene. The relative positions of the TATA box, UAS, CP, *rbcSE9*, Nos Poly A site, *Kan^R^*, and Nos promoter and their respective cloning sites are shown where the arrow indicates the course of transcription.

The schematic map of promoter constructs BSV, SCBV, MMV, BM, MB, SM, and MS coupled with *uidA* gene and other essential components such as *rbcSE9*, Nos PolyA, *Kan^R^* gene, and Nos promoter from the left to the right border of the T-DNA region is presented in **Figure 1**. The respective positions of UAS, CP, and TATA box were also depicted.

The above promoter constructs were transiently expressed in tobacco (*N. tabacum* and *N. benthamiana*) leaves and rice (*O. sativa*) and pearl millet (*Pennisetum glaucum*) seedlings as described in Materials and Methods. Their activities were compared to the ZmUbi1 promoter in monocot plants (rice and millet) and the CaMV35S promoter in dicot plants (tobacco).

**Figure 2:**
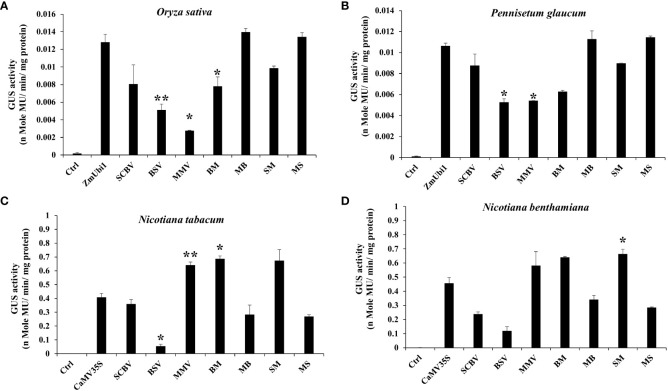
Transient GUS expression analysis of BSV, SCBV, MMV, BM, MB, SM, and MS promoters in **(A)** *Oryza sativa* (rice) and **(B)** *Pennisetum glaucum* (pearl millet) seedlings along with the ZmUbi1 promoter and **(C)** *N. benthamiana* and **(D)** *N. tabacum* leaves along with the CaMV35S promoter. *p ≤ 0.05; **p ≤ 0.01.

In rice and millet seedlings, the MB and MS chimeric promoters were efficient and had activities equivalent to the ZmUbi1 promoter in both plants (monocots) (**Figures 2A, B**). The chimeric promoters BM and SM showed lower expression in monocots. The overall strength of these promoters in monocots was in the following order: MB ≥ MS ≥ ZmUbi1 > SM > SCBV > BM > BSV > MMV. We observed that the activity of MB was 1.78–1.79 times stronger than BM in monocot. In addition, the activity of MS was 1.27–1.36 times stronger than SM in monocot.

In the case of tobacco plants (*N. tabacum* and *N. benthamiana*), contrary to the monocots, the chimeric promoters BM and SM were very efficient and showed higher activities than the CaMV35S promoter (**Figures 2C, D**). Comparatively, MB and MS showed weaker expression in dicot plants. The overall strength of these promoters in dicots was in the following order: SM ≥ BM ≥ MMV > CaMV35S > SCBV > MB > MS > BSV. The activity of BM was 1.8–2.42 times stronger than that of MB in dicot. Likewise, we observed that the activity of SM was 2.32–2.5 times stronger than that of MS in dicot.

### Expression analysis of chimeric promoters in callus

**Figure 3:**
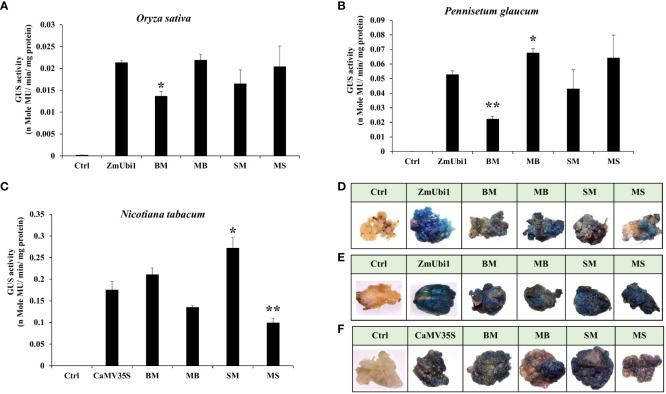
GUS expression analysis of hybrid promoters BM, MB, SM, and MS in regenerated callus of **(A)** rice and **(B)** pearl millet along with the ZmUbi1 promoter and **(C)** tobacco along with the CaMV35S promoter. Histochemical GUS staining of the transformed **(D)** rice, **(E)** pearl millet, and **(F)** tobacco calli was also represented. *p ≤ 0.05; **p ≤ 0.01.

The callus system provides an easier and more efficient method for comparative analysis of promoters *in planta*. Calli from tobacco, rice, and pearl millet were generated as described in Materials and Methods. We observed the activity of chimeric promoters SM, MS, BM, and MB in the following order: MB ≥ MS ≥ ZmUbi1 > SM > BM in both rice and pearl millet callus (**Figures 3A, B**) and SM > BM > CaMV35S > MB > MS in tobacco callus. The GUS activity for each construct was measured in triplicate and the activity was presented with the respective ± SD in **Figure 3C**.

The above result suggested that the MB and MS hybrid promoter showed better activity in rice and millet callus and was equivalent to the ZmUbi1 promoter, whereas the SM and BM promoter showed stronger activity than the CaMV35S promoter in tobacco callus.

These observations were also supported by X-Gluc staining of rice, millet, and tobacco calli, where the intensity of blue coloration was directly proportional to the transcriptional activity of the chimeric promoters as presented in **Figures 3D–F**, respectively.

### Transgenic analysis of chimeric promoters in tobacco plants

Approximately 10 transgenic tobacco plants expressing chimeric promoters BM, MB, SM, and MS, along with the CaMV35S promoter coupled to the GUS reporter gene, were raised as described in Materials and Methods. All the transgenic lines were checked for proper integration of the T-DNA region through gene integration analysis of the promoter, *uidA*, *rbcSE9*, and *nptII* genes, and an appropriate chi-square value with a 3:1 segregation ratio of kanamycin-resistant to kanamycin-sensitive (Kan^R^: Kan^S^) seedlings. Among the 10 independent lines of the above promoter constructs, BM (line 5), MB (line 5), SM (line 7), and MS (line 2) were chosen for further experimental analysis.

**Figure 4:**
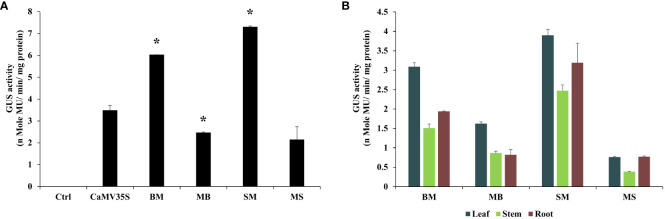
Transgenic analysis. Comparative GUS expression analysis of BM, MB, SM, MS, and CaMV35S promoters from **(A)** 21-day-old seedlings and **(B)** 45-days-old leaf, root, and stem tissue of T_2_ generation transgenic *N. tabacum* cv. Samsun NN plants was performed. *p ≤ 0.05.

Comparative analysis between different promoters was evaluated using 21-day-old T_2_ generation tobacco seedlings. It was found that the BM and SM promoters were highly active in transgenic tobacco plants and were 1.72 and 2.1 times stronger than the CaMV35S promoter, respectively. The MB and MS promoters showed moderate expression efficiency, approximately 0.7 and 0.61 times that of the CaMV35S promoter. Overall, in transgenic tobacco plants, the activities of chimeric promoters were in the following order: SM > BM > MB > MS, as presented in **Figure 4A**.

The GUS activity was measured from different plant tissues, such as leaf, stem, and root, of 45-day tobacco seedlings, and the average was evaluated in triplicate. The average transcriptional activities of these promoters are presented along with their respective standard deviation in **Figure 4B**. The data obtained revealed that the BM and SM promoters had higher expression in the leaf tissue and showed the following order of expression: Leaf > Root > Stem. However, the MB and MS promoters showed higher expression in the leaf but low expression levels compared to BM and SM.

**Figure 5:**
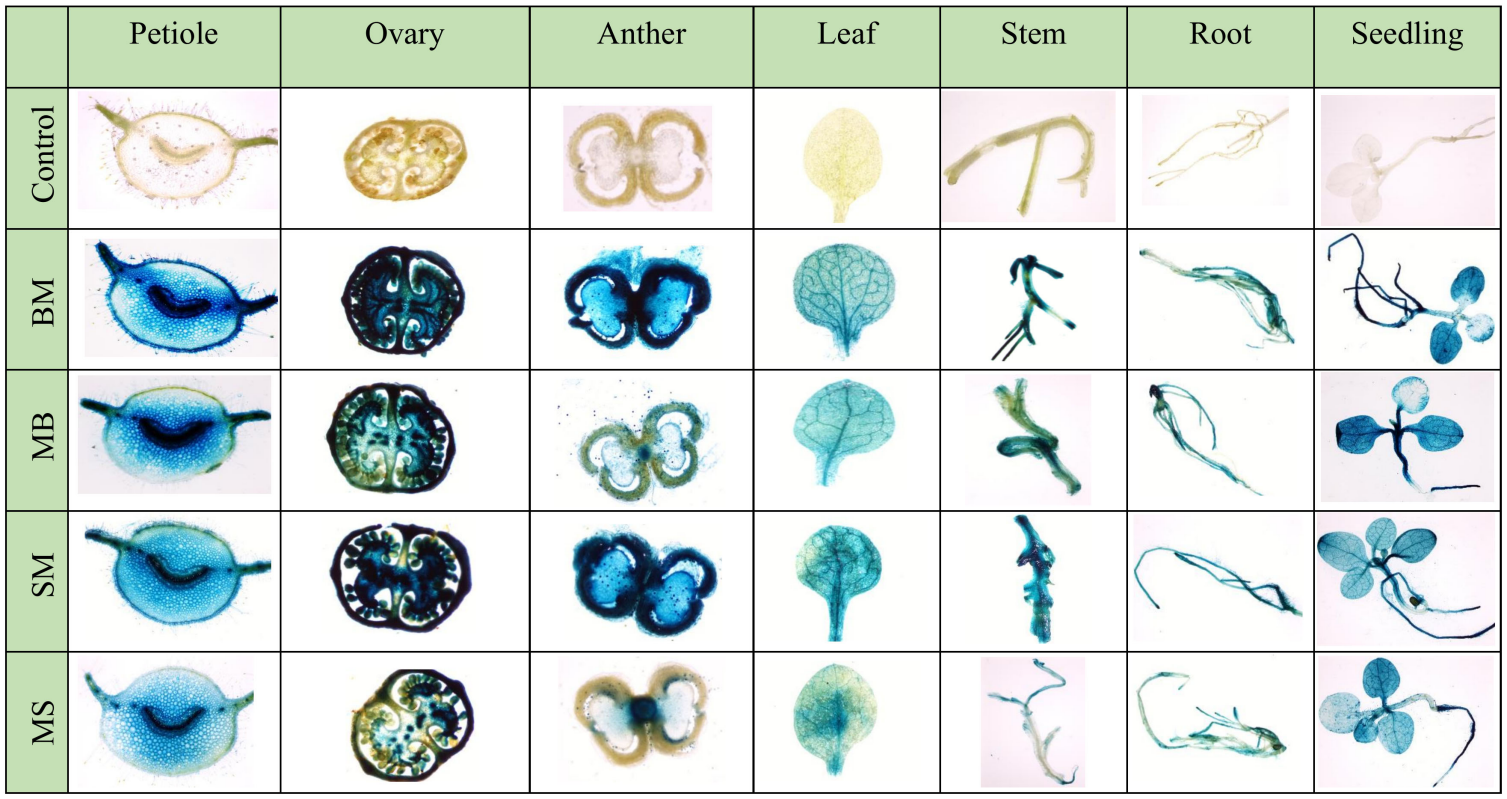
Histochemical GUS staining of different vegetative and reproductive parts of BM, MB, SM, and MS promoters containing transgenic tobacco plants.

Furthermore, the X-Gluc staining of vegetative (leaf, root, and stem) and reproductive parts (petiole, ovary, and anther) of T_2_ generation tobacco plants expressing these promoters showed high GUS expression in all the tissues, revealing the constitutive nature of these promoters (**Figure 5**).

### Expression analysis of deletion-hybrid promoter constructs

**Figure 6:**
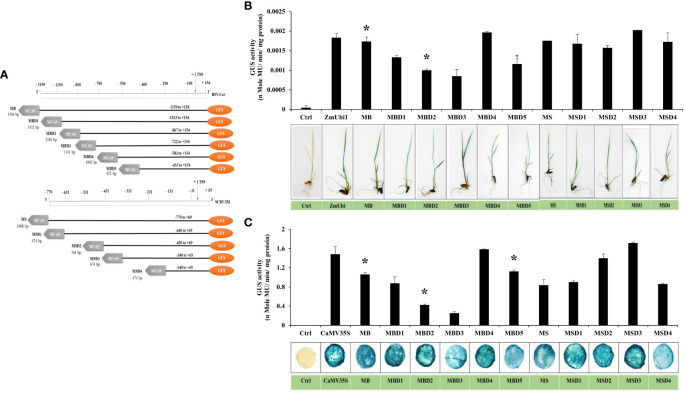
Analysis of deletion-hybrid promoters, MBD1, MBD2, MBD3, MBD4, MBD5, MSD1, MSD2, MSD3, and MSD4. **(A)** A schematic deletion map of promoter constructs with their coordinates fused to the GUS reporter gene. Transient GUS expression analysis of deletion fragments with their respective histochemical tissue staining in **(B)** rice seedlings and **(C)** tobacco leaves. *p ≤ 0.05.

Since not a single chimeric promoter showed high dual activity in the monocot and dicot systems, sequential deletion of SCBV and BSV fragments from MS and MB promoters was performed, to develop deletion promoters MSD1, MSD2, MSD3, MSD4, MBD1, MBD2, MBD3, MBD4, and MBD5 as described in Materials and Methods (**Figure 6A**). The promoter constructs were transiently expressed in *N. benthamiana* leaves and rice seedlings and kept for 48 h. The GUS activity was measured using fluorometric GUS assay as described in Materials and Methods, and the data from three independent events were measured and presented in **Figures 6B, C** with its respective ± SD. The plant tissue was also stained with X-Gluc solution, photographed, and presented in **Figures 6B, C**.

The result showed that all the deletion hybrid fragments were active in rice seedlings and tobacco leaves. In the case of the MB deletion fragments, we observed that MBD1, MBD2, and MBD3 had lower expression than the MB promoter in both rice and tobacco, whereas MBD4 had higher expression in both, while the MBD5 promoter had an equivalent expression to MB. In the case of MS deletion fragments, we observed that MSD1, MSD2, and MSD4 promoters were equivalent to MS promoters in rice seedlings, and MSD3 showed higher expression, whereas, in tobacco, MSD2 and MSD3 showed higher expression. The MSD3 and MBD4 promoters were 1.15 and 1.07 times stronger than the CaMV35S promoter in tobacco, and 1.1 and 1.07 times stronger than ZmUbi1 in rice, respectively. MSD3 from MS deletion and MBD4 from MB deletion showed the highest transcriptional activity in both rice and tobacco leaves.

**Figure 7:**
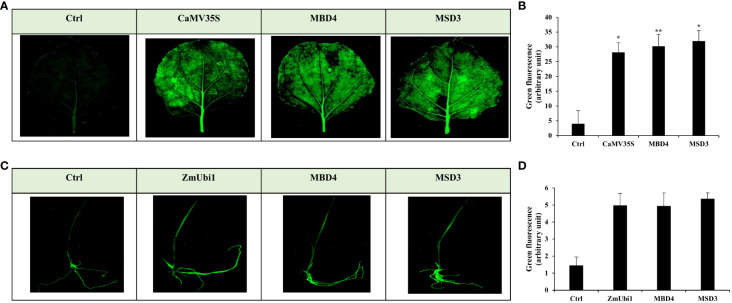
Transient GFP expression analysis of MBD4 and MSD3 promoters in agro-infiltrated **(A, B)** tobacco leaf and **(C, D)** rice seedling with its respective florescence intensity measured using ImageJ. *p ≤ 0.05; **p ≤ 0.01.

Furthermore, GFP expression analysis of MSD3 and MBD4 promoters was also performed in tobacco leaves and rice seedlings. The infiltrated tissue showed strong green coloration compared to the control (vector control), indicating high GFP accumulation (**Figure 7**).

### Expression analysis of MBD4 and MSD3 in tobacco, rice, and millet callus

**Figure 8:**
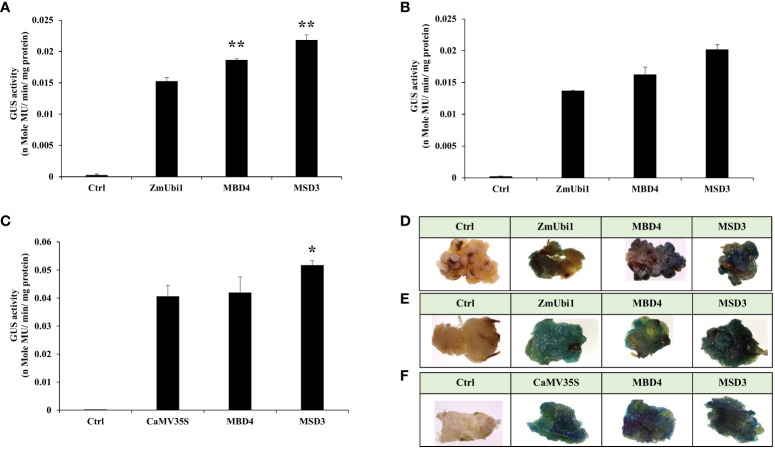
GUS expression analysis of MBD4 and MSD3 promoters in the regenerated callus of **(A)** rice and **(B)** pearl millet along with the ZmUbi1 promoter and **(C)** tobacco along with the CaMV35S promoter. Histochemical GUS staining of the transformed **(D)** rice, **(E)** pearl millet, and **(F)** tobacco calli was also represented. *p ≤ 0.05; **p ≤ 0.01.

The expression of MSD3 and MBD4 promoters was further validated in tobacco, rice, and pearl millet callus. The callus of these plants was generated and transformed with respective promoter constructs as described in Materials and Methods. The average GUS activity is presented in **Figures 8A–C** and its respective SD is calculated. Moreover, a few calli transformed with the above promoter constructs were stained with X-Gluc solution and photographed.

The result showed that the promoter MSD3 had high expression in transformed rice, millet, and tobacco callus. The MSD3 promoter showed higher expression than ZmUbi1 in rice and pearl millet callus and higher expression than the CaMV35S promoter in tobacco callus. The X-Gluc-stained transformed calli with MSD3 showed intense staining, suggesting a high gene expression level under the control of the MSD3 promoter in both monocot and dicot plants, as shown in **Figures 8D–F**.

## Discussion

The advent of plant synthetic biology has opened up a vast potential for new discoveries and inventions. Developing new, improved genetic toolkits allows more precise and efficient gene expression, which will be very useful for improving important traits in plants. A promoter plays an important role in such toolkits, as it is the key module for gene expression and regulation. A synthetic promoter offers an advantage over native promoters as it has more flexibility and can be developed to have specific features depending on its application (Sethi et al., 2021). These promoters can be developed either by tinkering with the cis-regulatory elements of already characterized native promoters or by completely developing new units using machine learning or directed evolution. These techniques have developed many synthetic promoters with unique features (Ranjan et al., 2012; Deb et al., 2018; Yang et al., 2021, Yang et al., 2022b; Yasmeen et al., 2023). However, even with all this progress, there are still some deficits in plant synthetic promoter collection. One of the most prominent limitations is being a single promoter with high gene expression in dicot and monocot plants. Still, now, different species-specific (dicot or monocot) promoters are being used for gene transformation in plants (Jiang et al., 2018). To reduce this burden, in this study, we have attempted to make a synthetic promoter with high gene expression in both the dicot and monocot plant systems.

For developing efficient synthetic promoters, domain hybridization of different promoters has successfully generated strong, constitutive promoters (Patro et al., 2012; Acharya et al., 2014b; Sherpa et al., 2023). Usually, an enhancer domain, also called UAS, is fused upstream of a native CP. The hybrid promoter generated is usually several-fold stronger than the native promoter. In our previous study, the native weakly expressing promoter HS4 from HRLV was hybridized with the UAS region of the FMV promoter (FUAS), and it led to the development of a strong synthetic promoter (FHS4), which was almost twofold stronger than its native promoter (Sherpa et al., 2023). The widely used strong constitutive promoter, the 35S enhanced, also called 2 × 35S or 35S^2^ promoter, is a by-product of domain hybridization of the 35S enhancer with the CaMV35S promoter (Kay et al., 1987; Acharya et al., 2014a; Deb et al., 2018; Amack and Antunes, 2020).

In this study, to develop a dual-species (monocot and dicot) expressing promoter, we hybridized the UAS region of two strong monocot-expressing promoters, SCBV and BSV, with the CP region of a strong dicot-expressing promoter, MMV, and vice versa. The UAS and CP regions were selected based on the highest expressing upstream and CP fragments of SCBV (Davies et al., 2014), BSV (Remans et al., 2005), and MMV (Dey and Maiti, 1999) promoters. The developed hybrid promoters, namely, BM, MB, SM, and MS, were transiently expressed in both monocot (rice and pearl millet) and dicot (*N. benthamiana* and *N. tabacum*) plants, and their activity was compared with the ZmUbi1 promoter in monocots and the CaMV35S promoter in dicots. In the case of native promoters, as expected, monocot-infecting pararetroviral promoters (SCBV and BSV) were more efficient in monocots than in dicots, and a dicot-infecting pararetroviral promoter (MMV) was more efficient in dicots than in monocots (**Figure 2**). Not surprisingly, the hybrids with monocot-specific CPs, viz., MB and MS, performed better in monocot systems and hybrids with dicot-specific CPs. BM and SM performed better in the dicot system. It was also interesting to note that the addition of MUAS in the case of hybrid MB and MS promoters greatly increased the activity of both BSV and SCBV promoters, especially in the monocot system, which was comparable to the ZmUbi1 in both rice and pearl millet seedlings (**Figures 2A, B**). Previous studies have also shown that MUAS is a very effective transcriptional enhancer when fused upstream of CPs (Deb et al., 2018; Sethi et al., 2022). Likewise, in the case of the dicot plants, the hybrid promoter with the dicot-specific CP, BM and SM, performed better in dicot plants than in monocot plants (**Figures 2C, D**).

Studying promoters in stable transgenic plants is undeniably one of the most reliable methods for its testing, but generating one is very time-consuming and labor-intensive. This is especially prevalent in monocot plants, which usually have long flowering cycles and are recalcitrant to different transformation techniques (Anjanappa and Gruissem, 2021). The callus system provides an efficient and simpler option for gene expression studies *in vivo*, especially for promoter comparative analysis. In this study, we generated callus of tobacco (*N. tabacum* cv. Samsun), rice (*O. sativa* IR64), and pearl millet (*Pennisetum glaucum*) and performed promoter activity analysis. We observed a similar pattern to transient analysis, where hybrids with dicot-specific CPs BM and SM performed well in tobacco callus, and hybrids with monocot-specific CPs MS and MB performed better in rice and millet callus (**Figure 3**).

Furthermore, to validate these results in stable transgenic plants, *N. tabacum* was transformed with promoters MB, BM, SM, MS, and CaMV35S, and their promoter activity analysis in different parts of transgenic plants was carried out. Consistent with the transient and callus expression data, the BM and SM were strong promoters. They showed higher expression than the MS and MB promoters (**Figure 4**). The histochemical staining of these transgenic plants suggested that BM and SM were constitutively expressed in all the parts of the plants, both vegetative and reproductive. In contrast, MS and MB showed semi-constitutive expression with high staining in leaf, root, stem, and ovary tissues and lighter staining in anther (**Figure 5**). Overall, the transgenic data correlated well with the transient and callus data regarding the comparative analysis of the promoters.

The generated chimeric promoters were species-specific and expressed higher in their respective system; therefore, a dual-expressing promoter could not be made. However, the MB and MS promoter worked well in monocots and had moderate expression (0.6–0.7 times that of CaMV35S) in dicots. We reasoned that since the MUAS enhancer is relatively farther from the CPs in MB and MS promoters, the effect of the enhancer might have been lower in dicots. To test this hypothesis, we sequentially deleted the 5′ region from BSV and SCBV and attached the MUAS region upstream of each fragment (**Figure 6A**). The deletion was done keeping in mind the placement of cis-regulatory element distribution in SCBV and BSV promoters. The deleted promoters were cloned and expressed into rice seedlings and tobacco leaves, and their respective promoter activity analysis was done (**Figure 6B**). Interestingly, the deleted-chimeric promoter MSD3 showed high expression efficacy in both rice and tobacco plants and could drive both *GUS* and *GFP* reporter genes efficiently (**Figure 6** and **Figure 7**). This difference was more noticeable in the callus system (**Figure 8**), where MSD3 was better than ZmUbi1 in rice and pearl millet callus and better than the CaMV35S promoter in tobacco callus. Different studies have shown that the position, distance, and combination of cis-regulatory elements in the promoter plays an essential role, either through direct protein–protein interactions or through protein–DNA interactions (Deplancke et al., 2016; Reiter et al., 2017; Cai et al., 2020). In our study, eliminating the −770 to −330 region of SCBV from the MS promoter led to the development of MSD3, which had high expression in both systems. We hypothesize that the phenomenon called passive cooperativity, a synergistic effect caused by a combination of cis-elements, led to the formation of a new enhanceosome complex, leading to higher expression in both dicot and monocot plants (**Supplementary Data 2**). Further study may be performed to understand this mechanism.

Taken together, we propose that the promoter MSD3 could be a valuable addition to synthetic plant promoter collection and will especially be useful in applications where high-level gene expression is required in both monocot and dicot systems.

## Conclusion

We performed intra-molecular domain hybridization between monocot-infecting pararetroviral-based promoter SCBV (coordinate: −770 to +69) and BSV (coordinate: −1,150 to +154) with a dicot-infecting pararetroviral-based promoter MMV (coordinate: −297 to +63) individually. The resultant chimeric promoters MB, BM, SM, and MS were found to be species-specific (dicot or monocot) based on the specific CP fragment. Furthermore, we designed and tested deletion-hybrid promoters using MB and MS promoters, which led to the characterization of a unique promoter, MSD3, with high-level gene expression (both GUS and GFP) in rice and tobacco plants. We conclude that the MSD3 promoter could be an important substitute for ZmUbi1 and CaMV35S promoters in plant biotechnology.

## Data availability statement

The original contributions presented in the study are included in the article/**Supplementary Material**. Further inquiries can be directed to the corresponding author.

## Author contributions

KK: Data curation, Formal analysis, Investigation, Methodology, Software, Validation, Visualization, Writing – original draft, Writing – review & editing. TS: Data curation, Formal analysis, Investigation, Methodology, Software, Validation, Visualization, Writing – original draft, Writing – review & editing. ND: Conceptualization, Funding acquisition, Investigation, Project administration, Resources, Supervision, Validation, Visualization, Writing – original draft, Writing – review & editing.
