# Supplementary material for: Analysis of plant pararetrovirus promoter sequence(s) for developing a useful synthetic promoter with enhanced activity in rice, pearl millet, and tobacco plants
Source: Front Plant Sci. 2024 Aug 6;15:1426479. doi: 10.3389/fpls.2024.1426479 (PMC11333926; doi:10.3389/fpls.2024.1426479)

**Supplementary Materials**

Analysis of plant pararetrovirus promoter sequence/s for developing useful synthetic promoter with enhanced activity in rice, pearl millet and tobacco plants

Khushbu Kumari**^a,b,^**^†^, Tsheten Sherpa**^a,b,^**^†^ , Nrisingha Dey**^a^**^*^

**Email:** **KK**: khusikumari357@gmail.com, **TS**: Tshetensherpa70@gmail.com, **ND**: nrisinghad@gmail.com

**Affiliation**

**^a^** Division of Plant Biotechnology, Institute of Life Sciences, NALCO Square, Chandrasekharpur, Bhubaneswar, Odisha 751023

**^b^** Regional Centre for Biotechnology, National Capital Region Biotech Science Cluster, Faridabad, Haryana (NCR Delhi) 121001

† These authors have contributed equally to this work

* Corresponding Author

Dr. Nrisingha Dey,

Email: nrisinghad@gmail.com

Phone: (+91)-674-2300728

Mobile: (+91)-9937163453

**Table S1:** Sequences of oligo primers for hybrid promoters’ development

| S.NO. | Primer name | Primer sequence |
| --- | --- | --- |
| 1 | 35S | **35S-F + 35S-R**  **35S-F** – 5’ ATGCGAATTCAGATCTCCTTTGCCCCAG 3’  **35S-R** - 5’ AGTGTCAAGCTTAGAGAGAGACTGGTG 3’ |
| 2 | ZmUbi1 | **ZmUbi1-F + ZmUbi1-R**  **ZmUbi1-F** - 5’ ATATGAATTCCCCGGGGCAGTGCAGCGT 3’  **ZmUbi1-R** - 5’ ATACAAGCTTCTGCAGAAGTAACACCAAACAACAG 3’ |
| 3 | SCBV | **SCBV-F + SCBV-R**  **SCBV-F** - 5’ GAGCCGTGAATTCGTCGACATTGAATGG 3’  **SCBV-R** - 5’ GTGCGGCAAGCTTTGCGGAAAGGTGTAATTC 3’ |
| 4 | BSV | **BSV-F + BSV-R**  **BSV-F** - 5’ ATATAGAATTCGTCGACGGTTGCATGGAAGG 3’  **BSV-R** - 5’ ATGATAAGCTTCCAAGGCTCTGATACCAGAAC 3’ |
| 5 | MMV-12 | **MMV-F + MMV-R**  **MMV-F** - 5’ ACTGATGAATTCGTCGA*C*TTCGTCCACAGAC 3’  **MMV-R** - 5’ ATCTAAAGCTTCCCGGGGGATTAGACGT 3’ |
| 6 | BUAS | **BSV-F + BUAS-R**  **BUAS-R** - 5’ ATATAAGCTTCCCGGGGAGGGAGTTCG 3’ |
| 7 | MUAS | **MMV-F + MUAS- R**  **MUAS-R** - 5’ ATCTAAAGCTTCCCGGGGGATTAGACGT 3’ |
| 8 | SUAS | **SUAS-F + SUAS-R**  **SUAS-F** - 5’ ATATTGAATTCGTCGACGAACACCGTTCGAG 3’  **SUAS-R** - 5’ GCTATAAGCTTCCCGGGCAAAGCATCGCATA 3’ |
| 9 | SUASMCP (SM) | **SUAS-F + MMV-R** |
| 10 | MUASSCP (MS) | **MMV-F + SCBV-R** |
| 11 | BUASMCP (BM) | **BSV-F +MMV-R** |
| 12 | MUASBCP (MB) | **MMV-F + BSV-R** |

**Table S2:** Sequences of oligo primers for the development of deletion-hybrid promoter constructs

| S.NO. | Construct | Sequence |
| --- | --- | --- |
| 1 | MSD1 | **MUAS-F + (SD1-F + SCBV-R)**  **SD1-F** – 5’ GAGCGCTGAATTCGTCGACCAATCATGTTAAAATC 3’ |
| 2 | MSD2 | **MUAS-F + (SD2-F + SCBV-R)**  **SD3-F** – 5’ ATATTGAATTCGTCGACACCGTTCGAGTGTC 3’ |
| 3 | MSD3 | **MUAS-F + (SD3-F + SCBV-R)**  **SD4-F** – 5’ ATTACGAATTCGTCGACAAGAGTGCCACACA 3’ |
| 4 | MSD4 | **MUAS-F + (SD4-F + SCBV-R)**  **SD5-F** – 5’ ATAACGAATTCGTCGACTGTGTGTGTCCTTTTG 3’ |
| 5 | MBD1 | **MUAS-F + (BD1-F + BSV-R)**  **BD1-F** – 5’ GCATGAATTCGTCGACAGTCATCAACAGCTTGGATAAAT 3’ |
| 6 | MBD2 | **MUAS-F + (BD2-F + BSV-R)**  **BD2-F** – 5’ GCATGAATTCGTCGACACTATATCACTGGAACAGGATTG 3’ |
| 7 | MBD3 | **MUAS-F + (BD3-F + BSV-R)**  **BD3-F** – 5’ GCATGAATTCGTCGACAGAAGAAATACTTCAGAAAGGA 3’ |
| 8 | MBD4 | **MUAS-F + (BD4-F + BSV-R)**  **BD4-F** – 5’ ATATGAATTCGTCGACATGTGGTTGCAGGAAACCT 3’ |
| 9 | MBD5 | **MUAS-F + (BD5-F + BSV-R)**  **BD5-F** – 5’ TAATGAATTCGTCGACGACCAAGTAAGCCAGTTGCC 3’ |

**Table S3:** Sequences of oligo primers for GUS, *nptII* and *rbcSE9*

| 1 | *GUS (uidA*) | **GUS F:** 5’ GATCGCGAAAACTGTGGAAT 3’  **GUS R**: 3’ TAATGAGTGACCGCATCGAA 5’ |
| --- | --- | --- |
| 2 | *nptII* (*Kan^R^)* | **Kan F**: 5’ ATGGCAATTACCTTATCCGCAACT 3’  **Kan R**: 3’ TCAGAAGAACTCGTCAAGAAGGCG 5’ |
| 3 | rbcSE9 | **rbsSE9 F**: 5’ GCGTCCGGATCCGCTTTCGTTCGTATCATCGGTTTC 3’  **rbcSE9 R**: 3’ ATGTAGTCTAGATGATGCATGTTGTCAATCAATTGG 5’ |
| 4 | *GFP* | **GFP F**: 5’ ATGGTGAGCAAGGGCGAG 3’  **GFP R**: 3’ TTACTTGTACAGCTCGTCC 5’ |

**Supplementary data 1:**

Sequences of few important constructs:

- TSS is represented as red coloured font

>**BSV-Cav (1304 bp) (-1150 to +154)**

**5’**GGTTGCATGGAAGGTTGGGGAGGAGTTTGTAAATGGAAAGAACAATCAGGACAACCAAGATGGTCAGAGAAGATTTGTGCTTATGCGAGTGGAAAGTTTAATCCGATCAAGAGCACAATTGATGCAGAAATTCAAGCAGTCATCAACAGCTTGGATAAATTCAAGATATATTATCTTGATAAAAAGGAGTTGATCATCAGGACGGATAGTCAAGCGATAGTCAGTTTCTACAAGAAGAGTAGTGACCACAAACCCTCAAGGGTAAGATGGTTAGCTTTCACTGACTATATCACTGGAACAGGATTGGATGTGAAGTTTGAGCATATTGACGGCAAGGATAATGTGCTAGCAGACACTCTGTCAAGGCTAGTAAAAATCATATGCCACAAGGAGAAACATCCATCAGAAACAATATTGATCAACGTTGCAGAAGAAATACTTCAGAAAGGAAGTATTGGAGCAAAAAGAAAGTTGGGAGAAATGATAAGTGGATATGAATCTTGGATGACAAGAATCCAAGAACACAAAATCAAGACACTAACACTTATCGAAAAACCAGTTTTTAAATGTGGTTGCAGGAAACCTGCTAGGCTTCACACGTCCAGGACATCAAGAAATCCGGGAAGAGAATTTTACTCATGTGAAAATAAAGCATGTTTCACTTGGGTATGGAAGGATCAGATTGATGAATACGTTCAAGAAGTGATGACGTGGAACGACCAAGTAAGCCAGTTGCCAGAAGAACCAGAAGGCTACAATGAAGGATGCACGATTGAAGACGCATTCGATCTGCTAGACGTCAGCAATGACGATCAATGGGCAAGGTCGTAAGCCATGACGTAGCGGAAGTGATGGACCCCATACCACTGGATGGCACTAACCAGTGTGACAAGGATACGAGATGCCAAGTGAGCTGGATAGCACTCACTTTATGTAAAGAGTGGTCTGCGTACCAACTCCACTATAGTCTGTCTGAGGTGCGATGCTGTGTCACGCACAAAGACTTTAGATTCCTTTGCGTGAGATGTACGCAAAGCAGTGTGTCCAGAGTGTGCTGTGACGCGTCCCTTGCATTATTGGTGGGTGCACCTAACGATGCGGGAAGCCGAACTCCCTCTATAAATAGGACCCCGTGTATTCAGTTGCAAGC**A**CGCAACACAACGCGAGCTTACTTCTGAGAAGAAATAAGAACAATTTGTGCTTGAAATACACCTTGTGTCAAGAGTGTGAGTAGAGCGCAAGATCCGTGTTGGGAAATCCGTGCCGTTCTGGAAATCCGTGCCGTTCTGGTATCAGAGCCTTGG **3’**

**>SCBV-IM (839 bp) (-770 to +69)**

**5’**AATCTTATTGAATGGGGAAAACAAATTCTTGATCCATTCCCCAAATTCAAGAAGGATATGTTTGAAAGAACTGAACATATCATGATGGCAACACAAGAGCCTACGCTACTATGTGGATGCAGGAAGCCTGCAATCATGTTAAAATCAGGAACAAGGCTTAATCCTCGTAGAAGATTTTACAAGTGTGCCATGAATATCTGCCACTGCTGGTATTGGGCAGATTTACTTGAAGAATACGTGCAAGAGAGGATCGAAGATTTCATGGTTGAAAACTTCGACAAGAAAGCAAAGCTGGATGAACCAAGTTCATCAAACGTTCACCATGATGATTATGAAGAACACCGTTCGAGTGTCATCGACAGGCCAAGGCCAACAGATGATCATTTCAGAACATGGGGGGATGTTACATACTGGCTGAATAAAGAAGCAGAAGAGTGCCACACAAGGGGCGACAACGTCGAAGGCGCAGAAGACGCAGTCGATCTCACTGACGTAAGCAATGACGACCAGTGGAGGAGATCGTAAGCAATGACGTATGGAGCGTGGAGGACCCATGAAAGCACTGAGAAGGCATCTCAACTTTCGGTGTGTGAGTGCGCATCCTATGCGATGCTTTGTACCTTTGTTAGCTGTGTGTGTCCTTTTGGCATCTGTGCCACTTTACCTTTGTCGGCCACGTTGCCTTTGCTTAGCATCTACGCAAGCATAGCGCTCGGCTGGTGTGTGTTCCCTCTGCCTATATAAGGCATGGTTGTATGACTCTTACACTC**A**TCGGTAGTTCACCACATGAGTATTTGAGTCAAGTTTGGCTTGAATAATAAGAATTACACCTTTCCGCA **3’**

>**MMV-12 (360 bp) (-297 to +63)**

**5’**TTCGTCCACAGACATCAACATCTTATCGTCCTTTGAAGATAAGATAATAATGTTGAAGATAAGAGTGGGAGCCACCACTAAAACATTGCTTTGTCAAAAGCTAAAAAAGATGATGCCCGACAGCCACTTGTGTGAAGCATGTGAAGCCGGTCCCTCCACTAAGAAAATTAGTGAAGCATCTTCCAGTGGTCCCTCCACTCACAGCTCAATCAGTGAGCAACAGGACGAAGGAAATGACGTAAGCCATGACGTCTAATCCCACAAGAATTTCCTTATATAAGGAACACAAATCAGAAG**G**AAGAGATCAATCGAAATCAAAATCGGAATCGAAATCAAAATCGGAATCGAAATCTCTCATCT **3’**

**>ZmUbi1 (1996 bp) [-899 to +1097, Mutation at +497 to +501]**

**5’**CTGCAGTGCAGCGTGACCCGGTCGTGCCCCTCTCTAGAGATAATGAGCATTGCATGTCTAAGTTATAAAAAATTACCACATATTTTTTTTGTCACACTTGTTTGAAGTGCAGTTTATCTATCTTTATACATATATTTAAACTTTACTCTACGAATAATATAATCTATAGTACTACAATAATATCAGTGTTTTAGAGAATCATATAAATGAACAGTTAGACATGGTCTAAAGGACAATTGAGTATTTTGACAACAGGACTCTACAGTTTTATCTTTTTAGTGTGCATGTGTTCTCCTTTTTTTTTGCAAATAGCTTCACCTATATAATACTTCATCCATTTTATTAGTACATCCATTTAGGGTTTAGGGTTAATGGTTTTTATAGACTAATTTTTTTAGTACATCTATTTTATTCTATTTTAGCCTCTAAATTAAGAAAACTAAAACTCTATTTTAGTTTTTTTATTTAATAATTTAGATATAAAATAGAATAAAATAAAGTGACTAAAAATTAAACAAATACCCTTTAAGAAATTAAAAAAACTAAGGAAACATTTTTCTTGTTTCGAGTAGATAATGCCAGCCTGTTAAACGCCGTCGACGAGTCTAACGGACACCAACCAGCGAACCAGCAGCGTCGCGTCGGGCCAAGCGAAGCAGACGGCACGGCATCTCTGTCGCTGCCTCTGGACCCCTCTCGAGAGTTCCGCTCCACCGTTGGACTTGCTCCGCTGTCGGCATCCAGAAATTGCGTGGCGGAGCGGCAGACGTGAGCCGGCACGGCAGGCGGCCTCCTCCTCCTCTCACGGCACGGCAGCTACGGGGGATTCCTTTCCCACCGCTCCTTCGCTTTCCCTTCCTCGCCCGCCGTAATAAATAGACACCCCCTCCACACCCTCT**T**TCCCCAACCTCGTGTTGTTCGGAGCGCACACACACACAACCAGATCTCCCCCAAATCCACCCGTCGGCACCTCCGCTTCAAGGTACGCCGCTCGTCCTCCCCCCCCCCCCCTCTCTACCTTCTCTAGATCGGCGTTCCGGTCCATGGTTAGGGCCCGGTAGTTCTACTTCTGTTCATGTTTGTGTTAGATCCGTGTTTGTGTTAGATCCGTGCTGCTAGCGTTCGTACACGGATGCGACCTGTACGTCAGACACGTTCTGATTGCTAACTTGCCAGTGTTTCTCTTTGGGGAATCCTGGGATGGCTCTAGCCGTTCCGCAGACGGGATCGATTTCATGATTTTTTTTGTTTCGTTGCATAGGGTTTGGTTTGCCCTTTTCCTTTATTTCAATATATGCCGTGCACTTGTTTGTCGGGTCATCTTTTCATGCTTTTTTTTGTCTTGGTTGTGATGATGTGGTCTGGTTGGGCGGTCGTTCTAGATCGGAGTAGAATTAATTCTGTTTCAAACTACCTGGTGGATTTATTAATTTTGGATCTGTATGTGTGTGCCATACATATTCATAGTTACGAATTGAAGATGATGGATGGAAATATCGATCTAGGATAGGTATACATGTTGATGCGGGTTTTACTGATGCATATACAGAGATGCTTTTTGTTCGCTTGGTTGTGATGATGTGGTGTGGTTGGGCGGTCGTTCATTCGTTCTAGATCGGAGTAGAATACTGTTTCAAACTACCTGGTGTATTTATTAATTTTGGAACTGTATGTGTGTGTCATACATCTTCATAGTTACGAGTTTAAGATGGATGGAAATATCGATCTAGGATAGGTATACATGTTGATGTGGGTTTTACTGATGCATATACATGATGGCATATGCAGCATCTATTCATATGCTCTAACCTTGAGTACCTATCTATTATAATAAACAAGTATGTTTTATAATTATTTTGATCTTGATATACTTGGATGATGGCATATGCAGCAGCTATATGTGGATTTTTTTAGCCCTGCCTTCATACGCTATTTATTTGCTTGGTACTGTTTCTTTTGTCGATGCTCACCCTGTTGTTTGGTGTTACTTCTGCAG **3’**

**>CaMV35S (965 bp)**

**5’**AGATCTCCTTTGCCCCAGAGATCACAATGGACGACTTCCTCTATCTCTACGATCTAGTCAGGAAGTTCGACGGAGAAGGTGACGATACCATGTTCACCACTGATAATGAGAAGATTAGCCTTTTCAATTTCAGAAAGAATGCTAACCCACAGATGGTTAAGAGGCTTACGCAGCAGGTCTCATCAAGACGATCTACCCGAGCAATAATCTCCAGGAGATCAAATACCTTCCCAAGAAGGTTAAAGATGCAGTCAAAAGATTCAGGACTAACTGCATCAAGAACACAGAGAAAGATATATTTCTCAAGATCAGAAGTACTATTCCAGTATGGACGATTCAAGGCTTGCTTCACAAACCAAGGCAAGTAATAGAGATTGGAGTCTCTAAAAAGGTAGTTCCCACTGAATCAAAGGCCATGGAGTCAAAGATTCAAATAGAGGACCTAACAGAACTCGCCGTAAAGACTGGCGAACAGTTCATACAGAGTCTCTTACGACTCAATGACAAGAAGAAAATCTTCGTCAACATGGTGGAGCACGACACGCTTGTCTACTCCAAAAATATCAAAGATACAGTCTCAGAAGACCAAAGGGCAATTGAGACTTTTCAACAAAGGGTAATATCCGGAAACCTCCTCGGATTCCATTGCCCAGCTATCTGTCACTTTATTGTGAAGATAGTGGAAAAGGAAGGTGGCTCCTACAAATGCCATCATTGCGATAAAGGAAAGGCCATCGTTGAAGATGCCTCTGCCGACAGTGGTCCCAAAGATGGACCCCCACCCACGAGGAGCATCGTGGAAAAAGAAGACGTTCCAACCACGTCTTCAAAGCAAGTGGATTGATGTGATATCTCCACTGACGTAAGGGATGACGCACAATCCCACTATCCTTCGCAAGACCCTTCCTCTATATAAGGAAGTTCATTTCATTTGGAGAGGACACGCTGAAATCACCAGTCTCTCTCT **3’**

**>SUASMCP (648 bp)**

**5’**GGAACACCGTTCGAGTGTCATCGACAGGCCAAGGCCAACAGATGATCATTTCAGAACATGGGGGGATGTTACATACTGGCTGAATAAAGAAGCAGAAGAGTGCCACACAAGGGGCGACAACGTCGAAGGCGCAGAAGACGCAGTCGATCTCACTGACGTAAGCAATGACGACCAGTGGAGGAGATCGTAAGCAATGACGTATGGAGCGTGGAGGACCCATGAAAGCACTGAGAAGGCATCTCAACTTTCGGTGTGTGAGTGCGCATCCTATGCGATGCTTTGCCCGACTTCGTCCACAGACATCAACATCTTATCGTCCTTTGAAGATAAGATAATAATGTTGAAGATAAGAGTGGGAGCCACCACTAAAACATTGCTTTGTCAAAAGCTAAAAAAGATGATGCCCGACAGCCACTTGTGTGAAGCATGTGAAGCCGGTCCCTCCACTAAGAAAATTAGTGAAGCATCTTCCAGTGGTCCCTCCACTCACAGCTCAATCAGTGAGCAACAGGACGAAGGAAATGACGTAAGCCATGACGTCTAATCCCACAAGAATTTCCTTATATAAGGAACACAAATCAGAAGGAAGAGATCAATCGAAATCAAAATCGGAATCGAAATCAAAATCGGAATCGAAATCTCTCATCT **3’**

**>MUASSCP (1104 bp)**

**5’**TTCGTCCACAGACATCAACATCTTATCGTCCTTTGAAGATAAGATAATAATGTTGAAGATAAGAGTGGGAGCCACCACTAAAACATTGCTTTGTCAAAAGCTAAAAAAGATGATGCCCGACAGCCACTTGTGTGAAGCATGTGAAGCCGGTCCCTCCACTAAGAAAATTAGTGAAGCATCTTCCAGTGGTCCCTCCACTCACAGCTCAATCAGTGAGCAACAGGACGAAGGAAATGACGTAAGCCATGACGTCTAATCCCCCGACAATCTTATTGAATGGGGAAAACAAATTCTTGATCCATTCCCCAAATTCAAGAAGGATATGTTTGAAAGAACTGAACATATCATGATGGCAACACAAGAGCCTACGCTACTATGTGGATGCAGGAAGCCTGCAATCATGTTAAAATCAGGAACAAGGCTTAATCCTCGTAGAAGATTTTACAAGTGTGCCATGAATATCTGCCACTGCTGGTATTGGGCAGATTTACTTGAAGAATACGTGCAAGAGAGGATCGAAGATTTCATGGTTGAAAACTTCGACAAGAAAGCAAAGCTGGATGAACCAAGTTCATCAAACGTTCACCATGATGATTATGAAGAACACCGTTCGAGTGTCATCGACAGGCCAAGGCCAACAGATGATCATTTCAGAACATGGGGGGATGTTACATACTGGCTGAATAAAGAAGCAGAAGAGTGCCACACAAGGGGCGACAACGTCGAAGGCGCAGAAGACGCAGTCGATCTCACTGACGTAAGCAATGACGACCAGTGGAGGAGATCGTAAGCAATGACGTATGGAGCGTGGAGGACCCATGAAAGCACTGAGAAGGCATCTCAACTTTCGGTGTGTGAGTGCGCATCCTATGCGATGCTTTGTACCTTTGTTAGCTGTGTGTGTCCTTTTGGCATCTGTGCCACTTTACCTTTGTCGGCCACGTTGCCTTTGCTTAGCATCTACGCAAGCATAGCGCTCGGCTGGTGTGTGTTCCCTCTGCCTATATAAGGCATGGTTGTATGACTCTTACACTC**A**TCGGTAGTTCACCACATGAGTATTTGAGTCAAGTTTGGCTTGAATAATAAGAATTACACCTTTCCGCA **3’**

**>MUASBCP (1569)**

**5’**TTCGTCCACAGACATCAACATCTTATCGTCCTTTGAAGATAAGATAATAATGTTGAAGATAAGAGTGGGAGCCACCACTAAAACATTGCTTTGTCAAAAGCTAAAAAAGATGATGCCCGACAGCCACTTGTGTGAAGCATGTGAAGCCGGTCCCTCCACTAAGAAAATTAGTGAAGCATCTTCCAGTGGTCCCTCCACTCACAGCTCAATCAGTGAGCAACAGGACGAAGGAAATGACGTAAGCCATGACGTCTAATCCCCCGACGGTTGCATGGAAGGTTGGGGAGGAGTTTGTAAATGGAAAGAACAATCAGGACAACCAAGATGGTCAGAGAAGATTTGTGCTTATGCGAGTGGAAAGTTTAATCCGATCAAGAGCACAATTGATGCAGAAATTCAAGCAGTCATCAACAGCTTGGATAAATTCAAGATATATTATCTTGATAAAAAGGAGTTGATCATCAGGACGGATAGTCAAGCGATAGTCAGTTTCTACAAGAAGAGTAGTGACCACAAACCCTCAAGGGTAAGATGGTTAGCTTTCACTGACTATATCACTGGAACAGGATTGGATGTGAAGTTTGAGCATATTGACGGCAAGGATAATGTGCTAGCAGACACTCTGTCAAGGCTAGTAAAAATCATATGCCACAAGGAGAAACATCCATCAGAAACAATATTGATCAACGTTGCAGAAGAAATACTTCAGAAAGGAAGTATTGGAGCAAAAAGAAAGTTGGGAGAAATGATAAGTGGATATGAATCTTGGATGACAAGAATCCAAGAACACAAAATCAAGACACTAACACTTATCGAAAAACCAGTTTTTAAATGTGGTTGCAGGAAACCTGCTAGGCTTCACACGTCCAGGACATCAAGAAATCCGGGAAGAGAATTTTACTCATGTGAAAATAAAGCATGTTTCACTTGGGTATGGAAGGATCAGATTGATGAATACGTTCAAGAAGTGATGACGTGGAACGACCAAGTAAGCCAGTTGCCAGAAGAACCAGAAGGCTACAATGAAGGATGCACGATTGAAGACGCATTCGATCTGCTAGACGTCAGCAATGACGATCAATGGGCAAGGTCGTAAGCCATGACGTAGCGGAAGTGATGGACCCCATACCACTGGATGGCACTAACCAGTGTGACAAGGATACGAGATGCCAAGTGAGCTGGATAGCACTCACTTTATGTAAAGAGTGGTCTGCGTACCAACTCCACTATAGTCTGTCTGAGGTGCGATGCTGTGTCACGCACAAAGACTTTAGATTCCTTTGCGTGAGATGTACGCAAAGCAGTGTGTCCAGAGTGTGCTGTGACGCGTCCCTTGCATTATTGGTGGGTGCACCTAACGATGCGGGAAGCCGAACTCCCTCTATAAATAGGACCCCGTGTATTCAGTTGCAAGCACGCAACACAACGCGAGCTTACTTCTGAGAAGAAATAAGAACAATTTGTGCTTGAAATACACCTTGTGTCAAGAGTGTGAGTAGAGCGCAAGATCCGTGTTGGGAAATCCGTGCCGTTCTGGAAATCCGTGCCGTTCTGGTATCAGAGCCTTGG **3’**

**>BUASMCP (1483 bp)**

**5’**GGTTGCATGGAAGGTTGGGGAGGAGTTTGTAAATGGAAAGAACAATCAGGACAACCAAGATGGTCAGAGAAGATTTGTGCTTATGCGAGTGGAAAGTTTAATCCGATCAAGAGCACAATTGATGCAGAAATTCAAGCAGTCATCAACAGCTTGGATAAATTCAAGATATATTATCTTGATAAAAAGGAGTTGATCATCAGGACGGATAGTCAAGCGATAGTCAGTTTCTACAAGAAGAGTAGTGACCACAAACCCTCAAGGGTAAGATGGTTAGCTTTCACTGACTATATCACTGGAACAGGATTGGATGTGAAGTTTGAGCATATTGACGGCAAGGATAATGTGCTAGCAGACACTCTGTCAAGGCTAGTAAAAATCATATGCCACAAGGAGAAACATCCATCAGAAACAATATTGATCAACGTTGCAGAAGAAATACTTCAGAAAGGAAGTATTGGAGCAAAAAGAAAGTTGGGAGAAATGATAAGTGGATATGAATCTTGGATGACAAGAATCCAAGAACACAAAATCAAGACACTAACACTTATCGAAAAACCAGTTTTTAAATGTGGTTGCAGGAAACCTGCTAGGCTTCACACGTCCAGGACATCAAGAAATCCGGGAAGAGAATTTTACTCATGTGAAAATAAAGCATGTTTCACTTGGGTATGGAAGGATCAGATTGATGAATACGTTCAAGAAGTGATGACGTGGAACGACCAAGTAAGCCAGTTGCCAGAAGAACCAGAAGGCTACAATGAAGGATGCACGATTGAAGACGCATTCGATCTGCTAGACGTCAGCAATGACGATCAATGGGCAAGGTCGTAAGCCATGACGTAGCGGAAGTGATGGACCCCATACCACTGGATGGCACTAACCAGTGTGACAAGGATACGAGATGCCAAGTGAGCTGGATAGCACTCACTTTATGTAAAGAGTGGTCTGCGTACCAACTCCACTATAGTCTGTCTGAGGTGCGATGCTGTGTCACGCACAAAGACTTTAGATTCCTTTGCGTGAGATGTACGCAAAGCAGTGTGTCCAGAGTGTGCTGTGACGCGTCCCTTGCATTATTGGTGGGTGCACCTAACGATGCGGGAAGCCGAACTCCCTCCCCGACTTCGTCCACAGACATCAACATCTTATCGTCCTTTGAAGATAAGATAATAATGTTGAAGATAAGAGTGGGAGCCACCACTAAAACATTGCTTTGTCAAAAGCTAAAAAAGATGATGCCCGACAGCCACTTGTGTGAAGCATGTGAAGCCGGTCCCTCCACTAAGAAAATTAGTGAAGCATCTTCCAGTGGTCCCTCCACTCACAGCTCAATCAGTGAGCAACAGGACGAAGGAAATGACGTAAGCCATGACGTCTAATCCCACAAGAATTTCCT**TATATAA**GGAACACAAATCAGAAGGAAGAGATCAATCGAAATCAAAATCGGAATCGAAATCAAAATCGGAATCGAAATCTCTCATCT **3’**

**Supplementary Data 2:**

a) Cis-distribution of MSD3 promoter


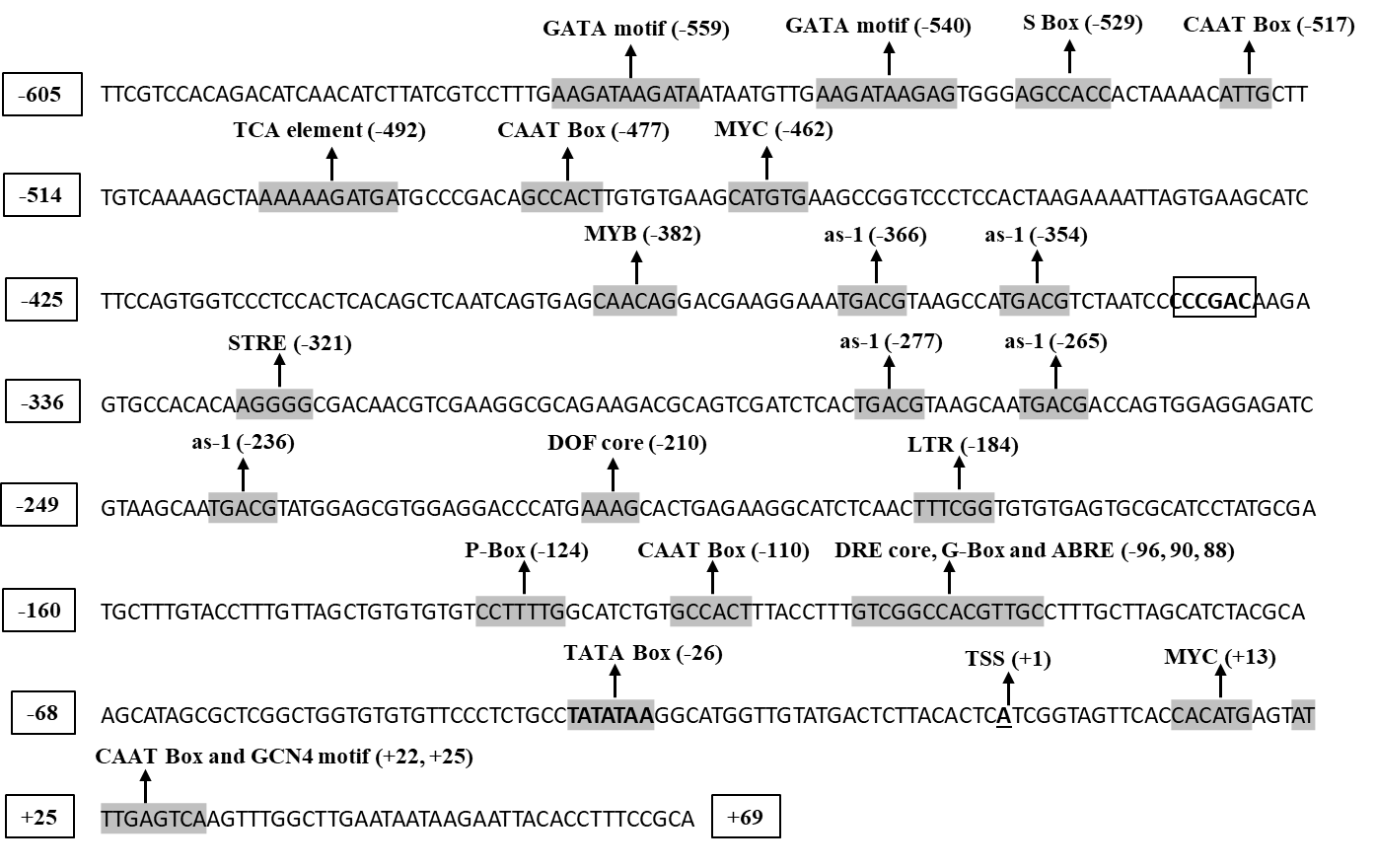


1. Cis-distribution of MBD4 promoter


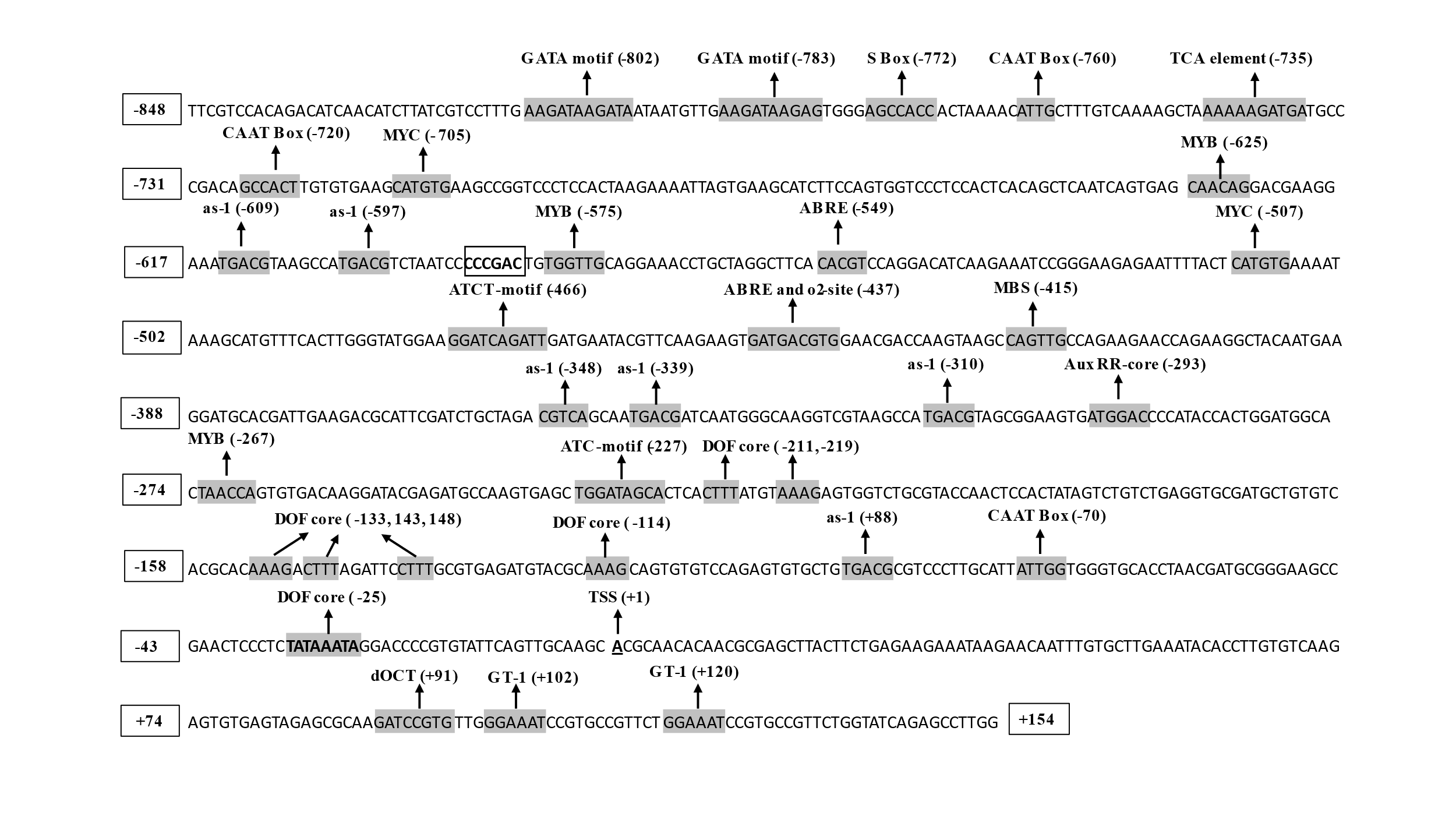

Supplement: Supplementary file 1 [file DataSheet_1.docx]
